# Distribution of *Helicobacter pylori *virulence markers in patients with gastroduodenal diseases in Pakistan

**DOI:** 10.1186/1471-230X-9-87

**Published:** 2009-11-20

**Authors:** Javed Yakoob, Shahab Abid, Zaigham Abbas, Wasim Jafri, Zubair Ahmad, Rashida Ahmed, Muhammad Islam

**Affiliations:** 1 Department of Medicine, Aga Khan University, Karachi, Pakistan; 2 Department of Pathology, Aga Khan University, Karachi, Pakistan

## Abstract

**Background:**

*Helicobacter pylori *(*H. pylori*) infection is known to be associated with a spectrum of gastroduodenal diseases. We studied the association of *H. pylori *virulence markers cytotoxin-associated gene (*cagA*) and vacuolating associated cytotoxin gene (*vacA*) alleles in patients with non ulcer dyspepsia (NUD), gastric ulcer (GU), gastric carcinoma (GC) and duodenal ulcer (DU).

**Methods:**

*H. pylori *infection established by both rapid urease test and histology were studied. The *cagA *and *vacA *allelic status was determined by polymerase chain reaction (PCR). Sequencing of *vacA i1 *and *i2 *PCR product was carried out.

**Results:**

Two hundred and twenty-four patients were included, 141 (63%) were males with a mean age of 45 ± 16, range 16-83 years. The virulence marker *cagA *was associated with GU in 20(63%) (p = 0.04), DU in 23(72%) (p = 0.003) and GC in 29(73%) (p = 0.001) compared to NUD in 51(42%). *VacA s1am1 *was associated with GU in 23(72%) (p = 0.001), DU in 17(53%) (p < 0.001) and GC in 23(58%) (p = 0.003) compared to NUD in 38(32%) while *vacA s1bm1 *was also associated with GU in 9(28%) (p = 0.001), DU in 12(37%) (p < 0.001) and GC 11(28%) (p < 0.001) compared to NUD in 13(11%), respectively. The diagnoses of GU in 21(66%), DU in 16(50%), GC in 20(50%) and NUD in 50(42%) were associated with moderately active chronic inflammation. *CagA *in 55(45%) (p = 0.037), *vacA s1am1 *in 51(51%) (P < 0.001), *s1bm1 *in 25(56%) (p = 0.002), *s1am2 *32(30%) (p < 0.001) and *s1bm2 *29(69%) (p = 0.004) were also associated with moderately active chronic inflammation.

**Conclusion:**

*CagA *was negative in majority of NUD patients with *H. pylori *infection. However, *cagA *was associated with peptic ulcer and GC. *VacA *allele's *s1am1 *and *s1bm1 *were associated with *H. pylori *associated diseases and inflammation.

## Background

*Helicobacter pylori *(*H. pylori*) infection leads to the development of chronic gastritis and may lead to the development of duodenal and gastric ulcers, gastric adenocarcinoma and lymphoma [1-3]. The prevalence of *H. pylori *is high in developing countries. Its seroprevalence in Pakistan exceeds 58% of our general population and is common in asymptomatic population [4] A recent study revealed an early colonization/infection of infants with *H. pylori *and a prevalence of 67% at 9 months of age in a peri-urban community in Karachi, Pakistan [5]. The prevalence varies among countries with existing evidence suggesting that the diversity in disease outcome may be ascribed to variations in infecting strains [6,7]. Two phenotypic characteristics among *H. pylori *strains, the high molecular weight protein encoded by the cytotoxin-associated gene A (*cag A*), and the vacuolating cytotoxin (*vac A*) have been found to be associated with distinct gastrointestinal disorders [8,9]. About 60-80% of *H. pylori *strains express the 120-to-140 kDa *cagA *product that is recognized by serum antibodies [10]. Various studies have demonstrated a strong association between the presence of antibodies to *cag A *and peptic ulcer disease and gastric carcinoma [11,12]. The gene encoding *vacA *is present in nearly all strains; however, the activity of this cytotoxin is positive in only 40-60% of patients with peptic ulcer disease and in 30% of *H. pylori *strains from patients with chronic gastritis [12,13]. The *vacA *gene present in all *H. pylori *strains comprises two variable parts, the '*s*' region (encoding the signal peptide) and two alleles, '*s1*' and '*s2*' have been recognized. Within type *'s1'*, several subtypes (*s1a*, *s1b *and *s1c*) can be distinguished [13]. For the '*m*' region (middle) two alleles, '*m1*' or '*m2*', have been identified. Recently, a novel determinant of *vacA *called the intermediate (*i*) region has been described [14]. It has been shown to be a better predictor of the carcinogenic potential of the *H. pylori *strains than the current signal region and midregion typing systems. The aim of this study was to investigate the distribution of tissue *cagA *and *vacA *allelic status in *H. pylori *positive gastroduodenal diseases and their associated histopathological changes in gastric mucosa.

## Methods

### Patients

Two hundred and twenty-four patients were included in the study. All patients were reported positive for *H. pylori *infection by the rapid urease test and or histology. There were 141 (63%) males and 83 (37%) females with a mean age of 45 ± 16, range 16-83 years. These patients presented with upper gastrointestinal symptoms and majority met the Rome III Diagnostic Criteria for Functional Dyspepsia i.e., at least 3 months, with onset at least 6 months previously, of 1 or more of the following: bothersome postprandial fullness, early satiation, epigastric pain or epigastric burning in the absence of structural disease to explain the symptoms [15]. The diagnosis in these patients was non ulcer dyspepsia (NUD) in 120(54%), gastric carcinoma (GC) in 40(18%), duodenal ulcer (DU) in 32(14%) and gastric ulcer (GU) in 32(14%). Of GC 22(15%) were in corpus, 13(33%) in antrum and 5(12%) in fundus. They were all adenocarcinomas 20(50%) were diffuse and 20(50%) intestinal in nature. They attended the gastroenterology outpatient and endoscopy suite from June 2007 to December 2008. The study was approved by the Ethics Review Committee of Aga Khan University. All patients gave an informed consent for endoscopy and participation in the study. None of the patients had received antibiotics, acid reducing drugs such as H2-receptor antagonists, acid pump inhibitors, nonsteroidal anti-inflammatory drugs or bismuth compounds in the last 4 weeks. The clinical symptoms at the time of presentation and endoscopic findings were noted. Gastric biopsy specimens were taken from an area of inflammation in the antrum and corpus. Two biopsy specimens were removed for each of the rapid urease test (Pronto Dry), histology and polymerase chain reaction (PCR). Two gastric biopsy specimens were inserted into rapid urease test (Pronto Dry). Specimens for histology were dispatched in formalin while for PCR in 0.9% normal saline. The *cagA *PCR for 5' terminal and *vacA *alleles for the signal "*s"*, middle "*m*" and intermediate region "*i" *were analyzed.

### Rapid Urease test

Pronto Dry results were read in 30 minutes after sampling as directed by the manufacturer. The color change from yellow to pink was considered positive [16].

### Histology

Gastric biopsy specimens for histopathology were stained with Hematoxylin and eosin (H & E) stain for the detection of *H. pylori *and degree of gastritis. In doubtful cases, Giemsa staining was carried out to ascertain presence of *H. pylori*. The degree of gastritis as determined on H & E stain was scored in accordance with the Sydney system [17]. The presence of *H pylori *was determined by the positive rapid urease test and histology. All biopsy specimens for histological examination were fixed in 10% formalin, embedded in paraffin wax on the oriented edge, and cut into 5 μm thick sequential sections. All tissue sections were stained with hematoxylin and eosin for histological examination. The degree of acute and chronic inflammation, as well as the *H pylori *density was scored according to the updated Sydney system. The bacterial density was graded from 0 to 3 (0, absent; 1 to 3, from few and isolated bacteria to colonies). The infiltration of gastric mucosa by mononuclear cells and polymorphonuclear leucocytes, atrophy, and intestinal metaplasia were graded as follows: 0, none; 1, mild; 2, moderate; 3, marked. Chronic inflammation was defined according to an increase in lymphocytes and plasma cells in the lamina propria graded into mild, moderate or marked increase in density. Chronic active gastritis indicated chronic inflammation with neutrophilic polymorph infiltration of the lamina propria, pits or surface epithelium graded as 0 = nil, mild =< 1/3 of pits and surface infiltrated; moderate = 1/3-2/3; and marked => 2/3. Antrum and corpus gastritis were scored by total sum of grade of gastritis (mild = 1, moderate = 2, marked = 3 infiltration with lymphocytes and plasma cells) and activity of gastritis (mild = 1, moderate = 2, marked = 3 infiltration with neutrophilic granulocytes) either in the antrum or in the corpus, a maximum of a sum of 6 points for each individual patient. Atrophy was defined as the loss of inherent glandular tissue, with or without replacement by intestinal-type epithelium. For optimal histological evaluation, all gastric biopsy specimens included surface epithelium and muscularis mucosae. Lymphoid aggregates were defined as accumulations of lymphocytes and plasma cells without a germinal centre.

### DNA extraction from tissues

DNA was extracted from gastric tissue as described before [18]. Briefly, gastric tissue was homogenized to uniformity in 500 μl of sterile water and centrifuged at 12,000 × g for 3 minutes. 500 ul of lysis buffer (100 mM NaCl, 10 mM Tris-HCl [pH 8.0], 25 mM EDTA, 0.5% sodium dodecyl sulfate), and 10 μl of Proteinase K (10 mg/ml) was added. Incubation was carried out at 50°C for 20 h; this was followed by phenol-chloroform extraction and ethanol precipitation. The resulting pellet was allowed to dissolve in 40 μl of TE buffer (10 mM Tris-HCl [pH 7.4] and 0.1 mM EDTA [pH 8.0] for 20 h at 37'C. Samples were stored at -20°C before PCR amplification was performed. DNA content and purity was determined by measuring the absorbance at 260 nm and 280 nm using a spectrophotometer (Beckman DU-600, USA).

### Polymerase chain reaction

Amplification of *cag A *and *vac A *alleles by PCR was performed in a volume of 50 μl containing 10 mmol/L Tris-HCl (pH 8.3), 50 mmol KCl, 1.5-2.5 mmol/L MgCl_2_, 200 μmol/L deoxynucleoside triphosphates, 2 units Taq DNA polymerase (Promega) and 25 pmol of both forward and reverse primers (Table 1) used before [14,19,20] (synthesized by MWG Automatic synthesizer). PCR was performed in a Perkin Elmer 9700 thermal cycler. The amplification cycles for *cagA *and *vacA *alleles are given in Table 1. Positive and negative reagent control reactions were performed with each batch of amplifications. DNA from *H. pylori *strains ATCC 43504 (*vacAs1am1*, *cagA *positive), ATCC 51932 (*vacA s2m2*, *cagA *negative) and ATCC 43526 (*vacA s1bm1, cagA *positive) was used to define the accuracy of the *cagA *and *vacA*-*i *PCR and positive control DNA for *vacA-i *region *i*1 and *i*2 labeled as 97-67 and 97-72, respectively [14]. After PCR, the amplified PCR products were electrophoresed in 2% agarose gels containing 0.5% × Tris/acetate/ethylenediaminetetraacetic acid, stained with ethidium bromide, and visualized under a short wavelength ultraviolet light source.

**Table 1:** Oligonucleotide primers used in typing of *H. pylori cag A *and *vac A *alleles

Region Amplified	Primer designation	Primer sequence (5' to 3')	Size of PCR product	PCR cycles
**CagA**				
C-5'	D008	GGTCAAAATGCGGTCATGG	297-bp ^19^	1 cycle of 94°C for 5 min, 35 cycles of 94°C for 1 min, 55°C for 1 min and 72°C for 90 sec, 1 cycle of 72°C for 5 min
	R008	TTAGAATAATCAACAAACATCACGCCAT		
**Vac A alleles**				
S1a	SS1-F	GTCAGCATCACACCGCAAC	190-bp ^19^	1 cycle of 95°C for 5 min; 35 cycles of 95°C for 1 min, 52°C for 1 min and 72°C for 1 min; 1 cycle of 72°C for 5 min
	VA1-R	CTGCTTGAATGCGCCAAAC		
S1b	SS3-F	AGCGCCATACCGCAAGAG	187-bp ^19^	
	VA1-R	CTGCTTGAATGCGCCAAAC-		
S2	SS2-F	GCTAACACGCCAAATGATCC	199-bp^19^	
	VA1-R	CTGCTTGAATGCGCCAAAC		
m1	VA3-F	GGTCAAAATGCGGTCATGG	290- bp ^19^	
	VA3-R	CCATTGGTACCTGTAGAAAC3'		
m2	VA4-F	GGAGCCCCAGGAAACATTG	352- bp ^19^	
	VA4-R	CATAACTAGCGCCTTGCAC		
i1	Vac-F1	GTTGGGATTGGGGGAATGCCG	426- bp^14^	1 cycle of 95°C for 90 sec; 35 cycles of 95°C for 30 sec, 53°C for 60 sec and 72°C for 30 sec; 1 cycle of 72°C for 5 min
	C1R	TTAATTTAACGCTGTTTGAAG'		
i2	Vac-F1	GTTGGGATTGGGGGAATGCCG	432- bp^14^	
	C2R	GATCAACGCTCTGATTTGA		

### Sequence analysis of vacA i region PCR product and BLAST Query

The DNA fragments amplified by *vacA i*-region, type's *i*1 and *i*2 PCRs were purified by Qiagen quick PCR purification kit (Qiagen, USA) and sequenced using both the forward and reverse primers (Table 1) to verify that they represented truly the *H. pylori *vacuolating cytotoxin gene. Sequence analysis was performed by Macrogen (Seoul, South Korea). Sequence comparison was carried out using the Blast program and the GenBank databases.

### Statistical assessment

The statistical package for social science SPSS (Release 16, standard version, copyright^© ^SPSS; 2007) was used for data analysis. The descriptive analysis was done for demographic and clinical features. Results were presented as mean ± standard deviation for quantitative variables and number (percentage) for qualitative variables. Differences in proportion were assessed by using Pearson Chi square, Fisher exact or likelihood ratio test where appropriate. Non-parametric Kruskal-Wallis test was used to compare inflammatory score among diagnoses and virulence markers. P value less than 0.05 was considered as statistically significant.

## Results

The symptoms of the patients included abdominal pain 161(72%), weight loss 23(11%), vomiting 11(5%), bloating 9(4%), hematemesis 12(6%) and melena 4(2%). The endoscopic findings were gastritis in 120 patients with NUD (54%), gastric carcinoma (GC) in 40(18%), gastric ulcer (GU) in 32 (14%) and duodenal ulcer (DU) in 32 (14%) (Table 2). The bacterial density was grade 1 for 80 (36%), grade 2 for 126 (56%) and grade 3 for 18(8%) patients. Chronic inflammation was present in 60 (27%) and chronic active inflammation in 164 (73%).

**Table 2:** Distribution of cagA and vacA alleles in patients

	Diagnosis in patients n = 224				
**Virulence marker**	**Non ulcer dyspepsia** **n = 120**	**Gastric ulcer** **n = 32**	**Gastric carcinoma** **n = 40**	**Duodenal ulcer** **n = 32**	P value*

***CagA***					
Positive	51 (42)	20(62)	31(77)	23(72)	<0.001
Negative	69 (58)	12(38)	9(23)	9(28)	
***VacAs1a***					
Positive	79 (66)	25(78)	31(77)	19(59)	0.211
Negative	41 (34)	7(22)	9(23)	13(41)	
***VacAs1b***					
Positive	30 (25)	12 (37)	17 (42)	13 (41)	0.100
Negative	90 (75)	20(63)	23(58)	19 (59)	
***VacAm1***					
Positive	50 (42)	27(84)	29(72)	29(91)	<0.001
Negative	70 (58)	5(16)	11(28)	3(9)	
***VacAm2***					
Positive	74 (62)	15(47)	15(37)	12(37)	<0.01
Negative	46 (38)	17(53)	25(63)	20(63)	
***VacAs1am1***					
Positive	38 (32%)	23(72)	23(56)	17(53)	<0.001
Negative	82 (68%)	9(28)	17(44)	15(47)	
***VacAs1bm1***					
Positive	13 (11)	9(28)	11(28)	12(38)	<0.001
Negative	107 (89)	23(72)	29(82)	20(62)	
***CagA s1am1***					
Positive	22 (18)	11(34)	22(55)	11(34)	<0.001
Negative	98(82)	21(66)	18(45)	21(66)	
***CagA s1bm1***					
Positive	19 (16)	11(34)	7(18)	5(16)	0.01
Negative	101 (84)	21(66)	33 (82)	27(84)	
***vacAi***					
i1 and i2 positive	4(3)	0	2(5)	0	< 0.001
i1 and i2 negative	2(2)	2(6)	1(3)	0	
i1 positive	5(4)	27(85)	33(82)	27(84)	
i2 positive	109(91)	3(9)	4(10)	5(16)	

* Differences in proportion were assessed by using Pearson Chi square, Fisher exact or likelihood ratio test where appropriate.

### Correlation of *H. pylori *genotypes with diagnosis

The virulence marker *cagA *was significantly associated with peptic ulcer disease and GC (Table 2). It was positive in 31(77%) with GC, 20(62%) with GU, 23(72%) with DU compared to 51(42%) with NUD (p =< 0.001, 0.044 and 0.003 respectively). The *vacA allele m1 *was significantly positive in patients with GC 29(72%), 29(91%) in DU and 27(84%) in GU compared to only 50(42%) in NUD (p = 0.001, <0.001 and <0.001, respectively). The *H. pylori *genotype *cagAs1am1 *was associated with GC in 22(55%) with GC, 11(34%) in DU and 11(34%) in GU compared to 22(18%) in NUD (p < 0.001, 0.05 and 0.05, respectively). The *H. pylori *genotype *cagAs1bm1 *was associated with GC in 10(25%), 11(34%) in DU and 6(19%) in GU compared to 5(4%) in NUD (p < 0.001, <0.001 and 0.012, respectively). *VacA *type *i*1 was associated with GC in 33 (82%), GU in 27(85%) and DU in 27(84%) while *vacA *type *i*2 with NUD in 109 (90%) (Table 2). *VacAi1 *was associated with 17(85%) of the intestinal type of GC and 16(80%) of the diffuse type. All the GC arising in the fundus 5(100%), 12(92%) in the antrum and 16(73%) in the corpus were positive for *vacA i1*. The prevalence of mixed infections was indicated by *vacA *mixed genotype patterns observed in 39(17%) of the 224 subjects 12(30%) with GC, 11(28%) with GU, 10(26%) with DU and 6(16%) with NUD.

### Correlation of histological changes with diagnosis and *H. pylori *genotypes

**Figure 1:**
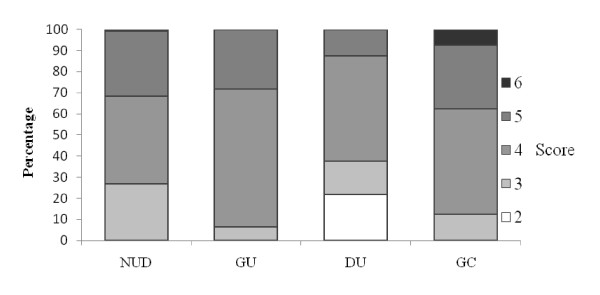
**Correlation of histological gastritis with diagnosis**. *NUD = non ulcer dyspepsia; *GU = gastric ulcer; *DU = duodenal ulcer; *GC = gastric carcinoma. *Gastritis score was a total sum of grade of gastritis (mild = 1, moderate = 2, marked = 3 infiltration with lymphocytes and plasma cells) and activity of gastritis (mild = 1, moderate = 2, marked = 3 infiltration with neutrophilic granulocytes), a maximum of a sum of 6 points for each individual patient. (Kruskal Wallis test; p = 0.005).

**Figure 2:**
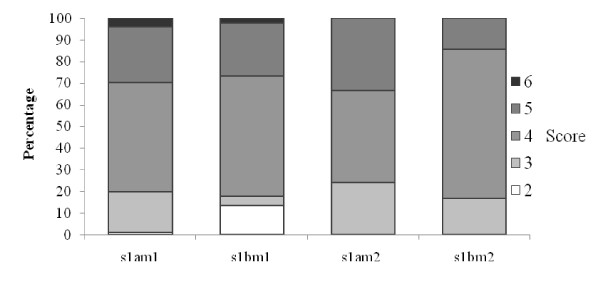
**Correlation of histological gastritis with H. pylori genotypes**. *Gastritis score was a total sum of grade of gastritis (mild = 1, moderate = 2, marked = 3 infiltration with lymphocytes and plasma cells) and activity of gastritis (mild = 1, moderate = 2, marked = 3 infiltration with neutrophilic granulocytes), a maximum of a sum of 6 points for each individual patient. (Kruskal Wallis test; p = 0.721).

**Figure 3:**
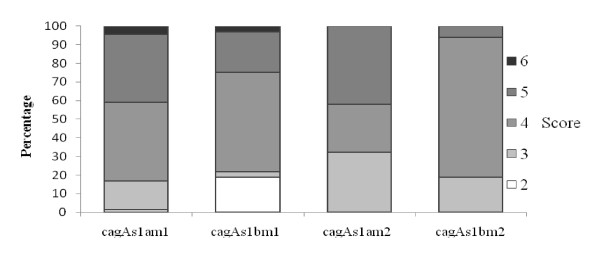
**Correlation of histological gastritis with H. pylori genotypes**. *Gastritis score was a total sum of grade of gastritis (mild = 1, moderate = 2, marked = 3 infiltration with lymphocytes and plasma cells) and activity of gastritis (mild = 1, moderate = 2, marked = 3 infiltration with neutrophilic granulocytes), a maximum of a sum of 6 points for each individual patient. (Kruskal Wallis test; p = 0.163).

Marked gastritis with a high inflammatory score was seen in patients with GC compared to NUD, GU and DU (Fig 1). A high degree of inflammation was seen associated with the *H. pylori vacA *genotypes *s1am1 *and *s1bm1 *compared to *s1am2 *and *s1bm2 *(Fig 2). Similarly, *cagAs1am1 *and *cagAs1bam1 *were associated with a higher degree of inflammation compared to *cagAs1am2 *and *cagAs1bm2 *(Fig 3). Intestinal metaplasia was demonstrated in 5(2%) cases, however, 3(60%) of these cases were associated with minimal inflammation with 2(2%) diagnosed as NUD and 1(3%) with GU (Fig 1). Gastric atrophy was demonstrated in 10(5%) in the body. Lymphoid aggregates were found in 107 (48%).

### Sequence analysis of vacA i region PCR product and BLAST Query

We sequenced PCR product of VacA *i*1 and *i*2 with Gene Bank accession number ACN25129, ACN25130, ACN25131, ACN25132 and ACN25133. Homology of the DNA sequences to published sequences was determined by using BLAST window on the National Center for Biotechnology Information (NCBI) site at http://www.ncbi.nlm.nih.gov/BLAST/. PCR product sequence aligned well with the sequences of vacuolating cytotoxin of different *H. pylori *strains e.g. ref [NC-000915.1] *Helicobacter pylori *26695, ref [NC-000921.1] *Helicobacter pylori *J99, ref [NC-008086.1] *Helicobacter pylori *HPAG1. We randomly selected three *vacAi*1 and two *vacAi*2 PCR products for sequencing. Of the three *vacAi*1 two were from GC (GenBank ACN25129, ACN25130) and one from a DU patient (GenBank ACN25131). The sequences of *i *region were almost identical to the *vacA *gene of strain 60190(GenBank U05676) except for seven mutations leading to amino acid substitution. Among these sequences, there were five conserved substitutions of N84K in cluster B (GTY***N***LSGL), G121S and S137L in cluster C (*G*ANRTTTRVDFNAKNI***S***ID), D171S and K194N in cluster D (TLQASEGITS***D***KNAEISLYDGATLNLASNSVKL*K*). In these strains from GC patients G -to-S, S-to-L and D-to-S substitutions were found. In strain from NUD patient with gastritis (GenBank ACN25133) in cluster A, K-to-Q; in cluster B R-to-I while in cluster C, A-to-T while S remained S and L remained L.

### Accession numbers

The sequences of two strains from GC and one from DU patient with an S-to-G substitution was deposited in GenBank (accession no. ACN25129, ACN25130, ACN25131). The strain from NUD patient with gastritis having substitution similar to GC and DU was deposited in the GenBank (accession no. ACN25132). The strain from NUD patient with gastritis for which the S remained S and L remained L was deposited in the GenBank (accession no. ACN25133).

## Discussion

The study of *H. pylori *virulence factor is important as it is associated with considerable morbidity and mortality. In Pakistan, infection with *H pylori *is frequent among general population and is acquired at an early age. It was found associated with dyspepsia previously in 66% of our patients [21]. The majority of our patients presenting with NUD had gastritis associated with *cagA *negative *H. pylori*. The associated *vac*A allelic types were *vacAs1am1 *and *vacAs1bm1 *(Table 2). On histology, gastric mucosa demonstrated both chronic and chronic active inflammation with or without lymphoid aggregate formation (Fig 2). *VacA i *region was not detected in 2% of the studied cases. There was a significant association of the *vacA *genotype *i*1 with GC, GU and DU while *vacA *type *i*2 with NUD (Table 2). In another study from the northwestern part of the country, Ahmad et al in a group of 78 dyspeptics with *H. pylori *gastritis described a prevalence of positive *cagA *in 24.2% [22]. The common *vacA *genotypes were *s1bm2 *in (54.2%) and *s1am1 *in (19.7%) [22]. This is in contrast to our study where the prevalence of *cagA *was 42% and *vacA *genotype *s1am1 *exceeded *s1bm1 *in patients with NUD. In keeping with previous study, only 10(5%) were detected with genotypes *vacAs2m2 *and no *s2m1 *was detected. Also, in our NUD patients, *cagA *was not significantly associated with *s1am1 *21(41%) (p = 0.201) and majority of *vacA *genotypes with *s1bm1*, *s1am2 *and *s1bm2 *were *cagA *negative. This higher prevalence of *cagA *may be explained by not only an increase number of patients in our study but also that our hospital is a tertiary care referral center with a large number of patients coming across from the different parts of our country.

This *H. pylori *virulence pattern in patients with peptic ulcer and GC were similar to the one described in Iran, India and Bangladesh [23-25]. A *cagA *prevalence of 76% was found in Iranian isolates [23]. PCR tests indicated that 80 to 90% of Calcutta strains carried the *cag *pathogenicity island (PAI) and potentially toxigenic *vacAs1 *alleles of the *vacA*, independent of disease status [24]. In Bangladesh, *cagA *was present in 75% of the strains from patients with peptic ulcer (PU) compared to 55% in patients with NUD, and 80% from PU carried potentially toxigenic *vacAs1 *alleles compared to 60% in isolates from patients with NUD [25]. However, no significant difference in any other virulence marker was observed in isolates from both groups. Phylogenetic analysis of the *vacA m *region and the 5' end of the *cagA *gene indicated that Bangladeshi isolates were more closely related to *H. pylori *isolates from India and were different from isolates from East Asia [25]. A relationship was described by Rhead et al between *i*1 and GC while Hussein et al finding no such relationship found *i*1 associated with GU in Iraq isolates [14,23]. Recently, Basso et al found an association of *i*1 not only with GC but also with DU in Italian isolates [26]. Our results concur with *i*1 association with GC, GU and DU.

## Conclusion

In our patients, the overall prevalence of *cagA *positive *H. pylori *infection is low, 56% compared to over 70% seen in neighboring countries, Iran, India and Bangladesh. It may possibly be explained by the lack of genetic predisposition and/or low frequency of particularly virulent *cagA *positive strain in this area. Karachi is a metropolitan city with people from different racial descent migrating to this part of the country may have resulted in the dilution of particularly virulent strains.

## Competing interests

The authors declare that they have no competing interests.

## Authors' contributions

JY conceived and designed the study, JY, SA, ZA, WJ coordinated the study, JY did the work, JY, ZA, RA, analyzed the data, JY and MI performed the statistical analysis. JY wrote the manuscript. All authors read and approved the final manuscript.

## Pre-publication history

The pre-publication history for this paper can be accessed here:

http://www.biomedcentral.com/1471-230X/9/87/prepub
